# Acknowledgement to Reviewers of Journal of Functional Morphology and Kinesiology in 2018

**DOI:** 10.3390/jfmk4010014

**Published:** 2019-01-28

**Authors:** Journal of Functional Morphology and Kinesiology Editorial Office

**Affiliations:** MDPI, St. Alban-Anlage 66, 4052 Basel, Switzerland

Rigorous peer-review is the corner-stone of high-quality academic publishing. The editorial team greatly appreciates the reviewers who contributed their knowledge and expertise to the journal’s editorial process over the past 12 months. In 2018, a total of 55 papers were published in the journal, with a median time to first decision of 19 days and a median time to publication of 47 days. The editors would like to express their sincere gratitude to the following reviewers for their cooperation and dedication in 2018:

Alferink, JudithFrizziero, AntonioArciero, PaulFulford, JonathanAssanelli, DeodatoGentil, PauloAziz, Abdul RashidGentile, PiergiorgioBalague Serre, NataliaGeorgian, BadicuBallmann, ChristopherGobbo, MassimilianoBarralon, PierreGonzalez, AdamBergdahl, Celena SchedeGoodla, LavanyaBest, RussGrenier, SylvainBianco, AntoninoGrey, Michael JamesBorer, Katarina T.Guglielmino, ClaudiaBrumitt, JasonHackney, MadeleineBurns, Ryan DonaldHaff, GuyCabri, JanHarper, Nicole G.Castrogiovanni, PaolaHassan, Amr AbdulfattahChaabene, HelmiHoriuchi, MasahiroCiccone, Marco MatteoHosick, PeterClark, Cain C THue, OlivierCoco, MarinellaIelapi, A.Conceicao, FIshihara, KengoCoratella, GiuseppeJensen, Eric D.Cortis, CristinaJoy, Jordan M.Costa, PabloKatsavelis, DimitriosCuadrado-Peñafiel, VíctorKellis, EleftheriosCuberos, Ramón ChacónKerhervé, HugoD’Amico, Agata GrazianaKingsley, J. DerekDeie, MasatakaKitrina, DouglasDickin, ClarkKovaleski, John E.Dillon, Edgar LicharKumar, GauravDUPREY, SONIALee, Han SukEarnest, ConradLee, KyuwanFeriche, BelénLoellgen, HerbertFernández-Revelles, Andrés B.Lopomo, Nicola FrancescoFontana, MarioLorenzetti, SilvioLouis, JulienRutkauskaite, RenataLowe, AndrewSarmento, HugoLugo, Ricardo GregorioSchmidt, SteffenLuis Ubago, JoseSeemann, EdgarLunn, WilliamSeibert, Franz JosefMamen, AsgeirSenchina, DavidManzano, RaquelSilva, LuisMartin, EricSinghal, KunalMascherini, GabrieleSoangra, RahulMason, LauraSoenen, StijnMcLeod, GeriSoles, GillianMethenitis, SpyridonSoundy, AndyMiotto, PaulaSousounis, KonstantinosMössner, MartinŠrámek, JanMusumeci, GiuseppeStefani, LauraNawrocka, AgnieszkaSubramanian, ArulNg, KwokSuchomel, TimothyNikolaidis, Pantelis TheodorosSzychlinska, MartaOcampo, DiegoTerzis, GerasimosOlek, RobertTimmerman, KyleOrzechowski, ArkadiuszTomczak, AndrzejOsier, NicoleTrovato, Guglielmo M.Oxford, SamTrue, LarissaPaschalis, VassilisVadalà, GianlucaPerciavalle, ValentinaValdes, AnaPerciavalle, VincenzoWagle, John P.Pitino, DarioWelter, Jean FredericPoston, BrachWillems, MarkRascher, DanielWoodfield, LorayneRauch, BernhardWorkman, VictoriaRavuri, SudheerYamagiwa, ShinichiRiebe, DeborahYano, TohruRoelofs, EricaZettel, JohnRuiz-Hincapie, PaulaZou, LiyeFontanella, Chiara GiuliaZubiaur, MartaForlenza, Samuel


